# Circulating Tissue Inhibitor of Matrix Metalloproteinase-4 (TIMP-4) in Systemic Sclerosis Patients with Elevated Pulmonary Arterial Pressure

**DOI:** 10.1155/2008/164134

**Published:** 2009-01-25

**Authors:** Elias J. Gialafos, Ioannis Moyssakis, Theodora Psaltopoulou, Dimitrios P. Papadopoulos, Despoina Perea, Kostantinos Vlasis, Charalampos Kostopoulos, Vassilios Votteas, Petros P. Sfikakis

**Affiliations:** ^1^Cardiology Department, Laikon General Hospital, University of Athens Medical School, 115 27 Athens, Greece; ^2^First Department of Propaedeutic and Internal Medicine, University of Athens Medical School, 115 27 Athens, Greece; ^3^Department of Hygiene and Epidemiology, University of Athens Medical School, 115 27 Athens, Greece; ^4^Pulmonary Unit, Department of Therapeutics, University of Athens Medical School, 115 27 Athens, Greece

## Abstract

Decreased levels of matrix metalloproteinases (MMPs) or excess levels of their tissue inhibitors (TIMPs) may contribute to dysregulation of extracellular matrix turnover in systemic sclerosis (SSc). In a cross-sectional study of 106 SSc patients, we measured serum levels of TIMP-4 which is preferentially expressed in cardiovascular structures and searched for correlations with simultaneously performed echocardiography measurements of pulmonary artery systolic pressure (PASP), myocardial performance, and pulmonary function tests. TIMP-4, but not MMP-9, levels were significantly raised in patients with SSc than controls. However, in the subgroup of patients with PASP measurements lower to 40 mmHg (*n* = 69), TIMP-4 levels were comparable to controls irrespective of the presence of diffuse or limited skin involvement, or lung fibrosis. Individual PASP measurements suggestive of pulmonary hypertension were associated with increased TIMP-4 serum levels (*P* = .03), independently of age, extent of skin sclerosis, or lung fibrosis, suggesting a cardiopulmonary vasculature-specific role of TIMP-4 activation in SSc.

## 1. INTRODUCTION

Systemic sclerosis (SSc) is characterized by excessive accumulation of collagen and other components of extracellular matrix in the skin and internal organs, being perhaps the prototypic disorder of a generalized disruption of connective tissue homeostasis [1]. Vasoconstriction and structural changes of the blood vessels, including intimal proliferation and obstruction, are expressed clinically as Raynaud's phenomenon, digital ulcers, renal disease, cardiac disease, and pulmonary hypertension (PH). Cardiopulmonary complications, including PH which occurs in a significant proportion of patients either as an isolated abnormality or secondary to pulmonary fibrosis, are currently the leading cause of death in SSc [1, 2]. Although effective screening for PH has proven difficult, many experts believe that early detection and intervention may alter the natural history of the disease [3].

Connective tissue turnover depends on the balance between the synthesis and degradation of the extracellular matrix. Extracellular matrix degradation is regulated mainly by matrix metalloproteinases (MMP-1 to MMP-28) and an important mechanism for the regulation of their activity is via binding to a family of homologous proteins, the tissue inhibitors of metalloproteinases (TIMP-1 to TIMP-4). Several lines of evidence indicate that the balance between MMPs and TIMPs levels governs connective tissue homeostasis, being a crucial determinant in inflammation, fibrosis and angiogenesis [4, 5]. Fibroblasts derived from patients with SSc produce increased amounts of TIMP-1, TIMP-2, and TIMP-3 [6, 7], whereas expression of MMP-1, MMP-2, and MMP-3 genes is decreased in fibroblasts from patients with early SSc compared to fibroblasts from healthy individuals or patients with late-stage disease [6]. These and other results suggest that excess levels of TIMPs, or decreased levels of MMPs may contribute to matrix accumulation in SSc.

TIMP-4 is the newest member in the mammalian TIMP family and differs from the other 3 TIMPs by its expression pattern. TIMP-4 is abundantly expressed in human cardiovascular structures, while all other tissues at the normal state, including the lung parenchyma, are characterized by low or absent expression [8]. Animal studies have suggested an important role of TIMP-4 in inflammatory diseases and cardiovascular pathologies [4, 5]. Moreover, TIMP-4 myocardial expression is remarkably increased in patients with aortic stenosis undergoing surgery [9], and in dilated cardiomyopathy patients with deteriorating heart failure [10]. On the other hand, MMP-9 is also found in cardiac myocytes, cardiac fibroblasts, and endocardial cells [4]. Although among other TIMPs there is only little specificity for inhibiting individual MMPs, key factors in every MMP inhibition are the size, charge, and polarity of residue 2 in the particular structure of TIMP-4 [11].

Based on the above, we hypothesized that aberrant TIMP-4 and/or MMP-9 activation may play a role in cardiovascular complications of SSc. To test this hypothesis, we examined serum levels of these molecules as well as of B-type natriuretic peptide (BNP), an established marker of SSc-related cardiovascular pathology [1, 12], and searched for correlations with echocardiography measurements of pulmonary artery systolic pressure (PASP), myocardial performance, and pulmonary function tests.

## 2. PATIENTS AND METHODS

### 2.1. Study population

One hundred and six consecutive patients (102 women) with SSc, aged between 22 and 80 years (mean 54 ± 13 years) and with disease duration ranging between 2 to 25 years (mean 11 ± 4 years) from date of the first non-Raynaud's phenomenon SSc manifestation, participated in this cross-sectional study. Blood samples were collected at the day of their regular follow-up which included lung function tests and echocardiography. SSc patients with previous myocardial infarction or stroke, valvular or congenital heart disease, hypertrophic cardiomyopathy, previous history of arterial hypertension, chronic obstructive pulmonary disease, or malignancies, as well those patients receiving bosentan or intravenous iloprost during the last 4 weeks, were excluded. Seventeen patients (15%) were current smokers. As shown in Table 1, medications included calcium-channel blockers in 77%, angiotensin-converting enzyme inhibitors in 55%, corticosteroids in 29%, cyclophosphamide in 22%, and mycophenolate mofetil in 7% of patients.

Pulmonary function tests (Master-Screen Diffusion, Jaeger, Wuerzburg, Germany) included spirometry, total lung capacity (TLC), and carbon monoxide diffusing capacity (DLCO) measurements, as described in [13, 14]. In all patients with both TLC and DLCO lower than 80% of predicted (indicative of pulmonary fibrosis) high resolution computed tomography of the lung [14] performed during the previous 6 months had confirmed the presence of fibrosis.

Complete echocardiographic examination (Hewlet-Packard Sonos 1000 ultrasound system, using a 2.5 MHz transducer) was performed as described in detail elsewhere [15], and established indices of myocardial performance for right and left ventricles (Tei-index) were calculated, as described in [16]. PASP was considered elevated when exceeded the level of 40 mm Hg [17]. There were 37 patients with elevated PASP; 18 of them had undergone right heart catheterization during the previous year confirming the presence of PH secondary to SSc [18] in all.

Sera collected from 24 age-matched healthy subjects (54 ± 19 years, 23 women), who fulfilled the exclusion criteria employed for patients, served as controls. All controls underwent a complete examination comprising electrocardiogram, echocardiography and exercise stress to exclude asymptomatic cardiac disease. Of the 24 control subjects, 8 women were current smokers, a marginally higher frequency comparing to 17 of 106 patients with SSc (*P* = .052). The study protocol was approved by Laikon Hospital and Alexandra Hospital ethics committees and all subjects gave informed consent.

### 2.2. Measurements of circulating TIMP-4, MMP-9, and BNP molecules

Circulating levels of TIMP-4 and MMP-9 were measured by quantitative sandwich enzyme-linked immunosorbent assays (Quantikine human TIMP-4 and Quantikine human MMP-9 total, respectively, R&D Systems Inc., Minneapolis, Minn, USA) according to the manufacturer's instructions in patient's and control sera that had been kept at −70 C. BNP concentrations were measured immediately after venipuncture in plasma samples from SSc patients using a sandwich immunoenzymatic assay (Triage BNP test, Biosite, San Diego, Calif, USA), according to the manufacturer's instructions.

### 2.3. Statistical analysis

Comparisons for continuous variables between groups were performed using *t*-test or Mann-Whitney test, in case of normal or skewed distribution, respectively. Age-adjusted partial correlation coefficients were built to evaluate examined correlations. TIMP-4, MMP-9/TIMP-4 ratios, and BNP were expressed as log10(TIMP-4), log10(MMP-9/TIMP-4), and log10(BNP), respectively, when correlated or regressed because of their skewed distribution. Bonferroni correction was used in cases of multiple testing to avoid false positive associations. Multivariate regression analysis was used to assess the association of TIMP-4 and/or MMP-9 levels with PASP, after controlling for possible confounders, such as age, presence or not of lung fibrosis, type of skin involvement (diffuse or limited), and BNP. The statistical package used was SPSS 13.0. Values are expressed as mean ± SD and a *P*-value < .05 was considered significant.

## 3. RESULTS

### 3.1. Circulating MMP-9 and TIMP-4 levels and extent of skin or pulmonary fibrosis in SSc

Of the 106 patients, 75 had diffuse (truncal skin involvement) and 31 patients had limited SSc (skin sclerosis confined to hands, arms, feet, and face), according to LeRoy's classification [19] (Table 1). Mean values of pulmonary function tests were comparable between patients with diffuse and limited SSc (Table 2); pulmonary fibrosis was present in 46 patients (34 with diffuse SSc).

**Figure 1:**
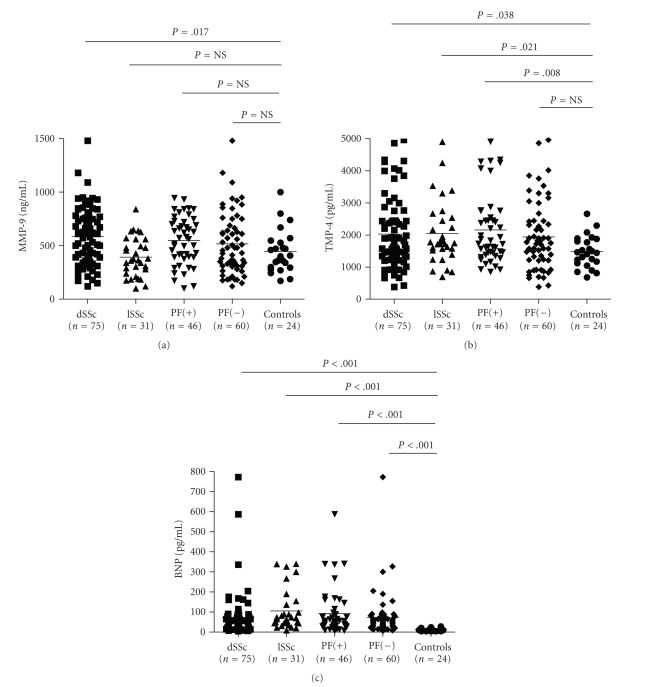
(a) MMP-9, (b) TIMP-4, and (c) BNP blood levels in SSc patients with diffuse (dSSc) and limited (lSSc) skin involvement as well as in those patients with pulmonary fibrosis (PF+) compared to healthy controls (Mann-Whitney test, NS denotes nonsignificant).

MMP-9 levels were not different between the whole SSc patient group and controls (530 ± 260 ng/mL versus 446 ± 201 ng/mL, resp.), but patients with diffuse SSc had higher MMP-9 levels than controls (Figure 1(a)) as well as than patients with limited SSc (587 ± 266 ng/mL versus 393 ± 182 ng/mL, *P* = .0003). No significant difference was noted between patients with lung fibrosis and those without (548 ± 222 ng/mL versus 517 ± 287 ng/mL).

In contrast to MMP-9, TIMP-4 levels were raised in the whole SSc patient group (2035 ± 1064 pg/mL, range 380–4961 pg/mL) compared to controls (1484 ± 489 pg/mL, range 683–2661 pg/mL) as well as in subgroups of patients with diffuse (2028 ± 1100 pg/mL, range 380–4961 ng/mL) or limited SSc (2050 ± 987 ng/mL, range 694–4900 ng/mL). Also, TIMP-4 levels were significantly higher in patients with pulmonary fibrosis (2157 ± 1068 ng/mL, range 846–4900 ng/mL) than controls (Figure 1(b)).

As shown in Table 3, age-adjusted partial correlations revealed no significant associations between individual serum levels of MMP-9, TIMP-4, or their ratio with corresponding pulmonary function tests. No significant differences, either for TIMP-4 or for MMP-9 mean levels, were noted between patients receiving, or not, angiotensin-converting enzyme inhibitors, or between patients receiving immunomodulatory drugs and the remaining patients.

### 3.2. Elevated pulmonary artery pressure in SSc is associated with increased TIMP-4 levels

Thirty seven of 106 patients with SSc (21 with diffuse and 16 with limited SSc) had PASP measurements equal or higher than 40 mm Hg in echocardiography (range 40–85 mm Hg, mean ± SD 51 ± 12 mm Hg). Age, disease duration, digital ulcers, arthritis, esophageal or intestinal involvement, antibodies to Scl-70, and current treatment regimens were comparable between patients with elevated PASP and the remaining patients. Of patients with elevated PASP, 22 had concomitant pulmonary fibrosis; the remaining 15 patients, all with limited SSc, had DLCO reduction as an isolated abnormality. As shown in Table 4, echocardiography-derived measurements of myocardial performance were significantly compromised in patients with elevated PASP measurements secondary to either diffuse or limited SSc, compared to the remaining patients. High BNP blood levels reflecting abnormalities in the cardiopulmonary vasculature were found in many of the studied patients with SSc (depicted in Figure 1(c)). As expected, BNP levels were increased by almost 5-fold in patients with elevated PASP than the remaining patients (Table 4).

**Figure 2:**
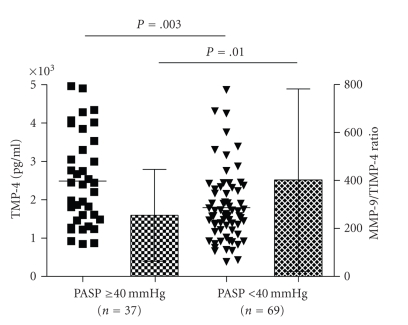
Patients with SSc and elevated pulmonary artery systolic pressure (PASP ≥ 40 mm Hg) have higher TIMP-4 levels and smaller MMP-9/TIMP-4 ratios (bars show SD) than the remaining patients (Mann-Whitney test).

As shown in Figure 2, TIMP-4 serum levels were considerably higher in SSc patients with elevated PASP measurements (2486 ± 1190 pg/mL, range 850–4961 pg/mL) than the remaining patients (1792 ± 909 pg/mL, range 380–4862 pg/mL, *P* = .003). Notably, after excluding the 37 patients with PASP ≥40 mm Hg from the whole SSc group, there were no significant differences in TIMP-4 levels between the control group and patients with diffuse (1767 ± 929 pg/mL, *n* = 53) or limited SSc (1875 ± 862 pg/mL, *n* = 16), or patients with lung fibrosis (1916 ± 918 pg/mL, *n* = 24), or those without (1726 ± 908 pg/mL, *n* = 45). TIMP-4 levels differed significantly between SSc patients, when as criterion for abnormally elevated PASP the level of 50 mm Hg (2880 ± 1174 pg/mL, *n* = 13, versus 1916 ± 998 pg/mL, *P* = .002), or the level of 45 mm Hg (2586 ± 1283 pg/mL, *n* = 23 versus 1882 ± 948 pg/mL, *P* = .02) was considered.

In contrast, MMP-9 serum levels were slightly lower in patients with elevated PASP than the remaining patients (511 ± 265 ng/mL and 541 ± 258 ng/mL, resp.). MMP-9/TIMP-4 ratios were significantly smaller in patients with elevated PASP (255 ± 192) than in those with PASP measurements lower than 40 mm Hg (402 ± 380) (Figure 2).

As shown in Table 3, age-adjusted partial correlation coefficients between individual TIMP-4 serum levels and the corresponding levels of PASP revealed a positive significant correlation. Since multiple testing may result to false positive associations, the Bonferroni correction was used, yielding the same results. On the other hand, echocardiographic indicators of either global myocardial performance (Tei-indices), or of left ventricle's systolic function (ejection fraction) did not correlate significantly with TIMP-4 circulating levels. In contrast, increased left ventricle Tei-index, indicative of impaired performance, was associated significantly with lower MMP-9 levels and MMP-9/TIMP-4 ratios (Table 3).

Finally, stepwise multivariate linear regression analysis was performed to assess possible associations among individual PASP measurements and the 3 corresponding clinical and laboratory parameters under study for the 106 SSc patients. Using this model, we found that increased PASP measurements were associated with TIMP-4 (log transformed-continuous, *β*-coefficient = 0.180, *P* = .031) and BNP (log transformed-continuous, *β*-coefficient = 0.534, *P* < .001). In contrast, no significant associations could be established with age (continuous), MMP-9 (continuous), SSc type (diffuse, limited), or presence of lung fibrosis (no, yes) in our SSc patient cohort.

## 4. DISCUSSION

In the present study, we found that TIMP-4 serum levels are increased in patients with either diffuse or limited SSc as well as in patients with pulmonary fibrosis. Because a relatively large number of patients were available, appropriate comparisons between patient subgroups were possible. No significant differences in TIMP-4 levels were noted between diffuse or limited skin involvement, or between patients with lung fibrosis and those without, suggesting a not convincing association of increased TIMP-4 serum levels with the extent of fibrosis characterizing SSc. However, further analysis showed that increased TIMP-4 circulating levels were higher in patients with elevated PASP measurements in echocardiography, irrespective of skin involvement extent or lung fibrosis. PASP was considered elevated when reaching or exceeding the level of 40 mm Hg in echocardiography, as also reported in other studies using noninvasive assessments of pulmonary pressure [15, 17, 20]. Clearly, echocardiography is not valid for the definite diagnosis of PH, but performing right cardiac catheterization in every patient was not possible. However, similarly significant associations between elevated PASP and TIMP-4 levels were also obtained when higher thresholds suggestive of PH, that is, 45 mm Hg [21, 22] or 50 mm Hg [18], were applied in our patient cohort.

Moreover, individual TIMP-4 levels correlated positively with the corresponding PASP measurements in our 106 patients with SSc. Treatment with angiotensin-converting enzyme inhibitors, known to influence TIMP-4 expression [23, 24], appeared not to affect this result. Age adjustment was applied in statistical analyses because MMP-9 may decrease [25], whereas TIMP-4 [25], BNP [26], PASP [17], and echocardiographic indices of myocardial performance [27] may increase with age. Finally, despite the limitation that serum measurements were performed only once, multivariate linear regression analysis revealed significant associations of PASP elevations and increases of TIMP-4 serum levels, but not with the presence of diffuse or limited SSc, or the presence of lung fibrosis in this cohort. In addition to TIMP-4, PASP was associated with increased BNP blood levels, as expected. Previous studies have shown that BNP levels are directly related to the severity of PH in SSc [12], and may be considered an independent predictor of PH in these patients [28].

To the best of our knowledge, no previous studies have examined TIMP-4 in patients with systemic connective tissue diseases or in patients with PH. Regarding TIMP-1 and TIMP-2 serum levels, both have been found elevated in SSc [29–34], and probably increased TIMP-2 levels correlate with cardiopulmonary complications [32, 33]. In a larger study examining both TIMP-1 and TIMP-2, only TIMP-1 levels were significantly elevated in diffuse and limited SSc compared to patients with primary Raynaud's phenomenon or controls, and no association with organ disease was found [34].

On the other hand, MMP-9 levels were significantly raised only in our subgroup of patients with diffuse SSc, in accordance with previous findings [35]. Overexpression of TGF-beta in scleroderma skin [36, 37] may contribute to local MMP-9 induction and proteolytic activation [38], thus resulting in increased circulating levels in patient with extended skin sclerosis. Such increases explain perhaps the trend toward significance of the inverse correlation of MMP-9 levels with DLCO and TLC measurements, since the majority of our patients with pulmonary fibrosis had diffuse SSc.

Patients with elevated PASP appeared to have lower MMP-9 mean levels, as also reported in SSc patients with PH [21]. Since bosentan [21] and iloprost [39] may attenuate MMP-9 expression, patients receiving such treatments were excluded from our study. Those patients with elevated PASP, most likely due to increased TIMP-4, had significantly smaller MMP-9/TIMP-4 ratios than the remaining patients, suggesting that different remodeling mechanisms of extracellular matrix may operate. Since MMP-9/TIMP-4 ratio reflects better the proteolytic activity, a decreased “net MMP activity” may favor decreased degradation of extracellular matrix components [40] within the cardiopulmonary vasculature in these patients. As shown in an experimental model of PH associated with marked inflammatory component [41], therapeutic inhibition of MMP activity by TIMP-1 gene transfer aggravated PH, indicating that MMPs play a protective role against pulmonary artery remodeling.

Moreover, lower individual MMP-9 levels and smaller MMP-9/TIMP-4 ratios were associated with impaired left ventricle myocardial performance, further implying a role of TIMP-4/MMP-9 interactions in cardiopulmonary vasculature abnormalities in SSc. Interestingly, cardiac remodeling in erythropoietin-transgenic mice, characterized by a stiffer left ventricle with diastolic dysfunction, is associated with decreased MMP-9 and increased TIMP-4 expression, followed by a shift in collagen mRNA expression from type III to type I [42]. It should be noted, however, that TIMPs and MMPs play also a complex role in regulating angiogenesis. For example, while TIMP-4 can induce apoptosis in cardiac fibroblasts [43], it may also act as an inhibitor of capillary endothelial cell migration, but not of proliferation or of angiogenesis in vivo [44]. On the other hand, mice hyperexpressing the profibrotic cytokine TGF-beta develop myocardial fibrosis and have a 2.5 increase of TIMP-4 myocardial expression compared to nontransgenic control mice [45].

## 5. CONCLUSION

The results presented herein may suggest that activation of TIMP-4, perhaps by leading to enhanced interactions with MMPs, plays a role in the increased stiffening within the cardiopulmonary vasculature in SSc. Whether this abnormality is a potential therapeutic target deserves further investigation. As reported recently, TIMP-4 gene was identified as one of 8 candidate genes for SSc in a pilot study using DNA pooling and genetic association analysis methods [46]. Prospective studies to examine whether serum TIMP-4 measurements may be used to identify high-risk SSc patients for cardiopulmonary complications, perhaps in combination with other biomarkers [3, 47], are warranted.

## Figures and Tables

**Table 1:** Characteristics of patients with systemic sclerosis (SSc).

	All patients	Diffuse SSc	Limited SSc
Women/men (*n*)	102/4	71/4	31/0
Mean age ± SD,	54 ± 13	52 ± 13	56 ± 12
years (range)	(22–80)	(22–80)	(25–77)
Mean disease duration ± SD,	11 ± 4	10 ± 3	12 ± 4
years (range)	(2–25)	(2–20)	(2–25)

* Medications,* % *of patients*(*n*)			

Calcium—channel blockers	77% (82)	76% (57)	81% (25)
Angiotensin—converting enzyme inhibitors	55% (58)	63% (47)	31% (11)
Corticosteroids	29% (31)	35% (26)	16% (5)
Cyclophosphamide	22% (21)	23% (17)	13% (4)
Mycophenolate mofetil	7% (7)	8% (6)	3% (1)

**Table 2:** Pulmonary function tests (mean ± SD of % predicted, *number of patients with less than 80*% *of predicted*) in patients with systemic sclerosis (SSc).

	All patients	Diffuse SSc	Limited SSc
	(*n* = 106)	(*n* = 75)	(*n* = 31)
FEV1	86 ± 17, 32	86 ± 18, 26	88 ± 16, 6
FVC	87 ± 19, 31	87 ± 20, 25	90 ± 16, 6
TLC	80 ± 16, 46	79 ± 16, 34	81 ± 17, 12
DLCO	68 ± 21, 81	67 ± 22, 54	69 ± 19, 27

FEV1: forced expiratory volume at 1 second; FVC: forced vital capacity; TLC: total lung capacity; DLCO: diffusing lung capacity for carbon monoxide.

**Table 3:** Age-adjusted partial correlations (*r*) of MMP-9, TIMP-4 and their ratio with respiratory and cardiac indicators in 106 patients with systemic sclerosis. Levels of significance are shown in parentheses.

	MMP-9	Log10(TIMP-4)	Log10(MMP-9/TIMP-4)
*Pulmonary function tests*		
* *FEV1 (%predicted)	−0.14 (NS)	−0.09 (NS)	−0.04 (NS)
* *FVC (%predicted)	−0.14 (NS)	−0.14 (NS)	−0.02 (NS)
* *TLC (%predicted)	−0.16 (*P* = .070)	−0.09 (NS)	−0.09 (NS)
* *DLCO (%predicted)	−0.19 (*P* = .068)	−0.70 (NS)	−0.11 (NS)

* Cardiac parameters*		

* *PASP	0.02 (NS)	0.29 (*P* = 0.021)	−0.15 (NS)
* *log10 (BNP)	0.00 (NS)	0.19 (NS)	−0.15 (NS)
* *RV Tei Index	0.02 (NS)	0.15 (NS)	−0.11 (NS)
* *LV Tei Index	−0.23(*P* = .046)	0.17 (NS)	−0.30(*P* = 0.006)
* *LV EF (%)	0.07 (NS)	−0.15 (NS)	0.15 (NS)

FEV1: forced expiratory volume at 1 second; FVC: forced vital capacity; TLC: total lung capacity; DLCO: diffusing lung capacity for carbon monoxide; PASP: pulmonary artery systolic pressure; BNP: B-type natriuretic peptide; RV: right ventricular; LV: left ventricular; EF: ejection fraction; NS denotes nonsignificant.

**Table 4:** Echocardiography-derived measurements of myocardial performance and BNP blood levels (mean ± SD) in SSc patients with normal or elevated PASP.

	RV Tei-index	LV Tei-index	LV EF (%)	BNP (pg/mL)
PASP ≤40 mm Hg (all patients, *n* = 69)	0.37 ± 0.02	0.38 ± 0.01	61 ± 9	33 ± 23
PASP >40 mm Hg (all patients, *n* = 37)	0.41 ± 0.03 (*P* < .001)*	0.41 ± 0.02 (*P* < .001)*	60 ± 5	163 ± 159 (*P*<.0001)*
PASP >40 mm Hg (diffuse SSc, *n* = 21)	0.41 ± 0.03	0.41 ± 0.02	60 ± 5	167 ± 187
PASP >40 mm Hg (limited SSc, *n* = 16)	0.42 ± 0.03	0.40 ± 0.02	61 ± 4	164 ± 120

SSc: systemic sclerosis; PASP: pulmonary artery systolic pressure; BNP: B-type natriuretic peptide; RV: right ventricular; LV: left ventricular; EF: ejection fraction. *Comparing to patients with PASP ≤40.

## References

[1] Denton CP, Black CM, Abraham DJ (2006). Mechanisms and consequences of fibrosis in systemic sclerosis. *Nature Clinical Practice Rheumatology*.

[2] Charles C, Clements P, Furst DE (2006). Systemic sclerosis: hypothesis-driven treatment strategies. *The Lancet*.

[3] Bull TM (2007). Screening and therapy of pulmonary hypertension in systemic sclerosis. *Current Opinion in Rheumatology*.

[4] Nagase H, Brew K (2003). Designing TIMP (tissue inhibitor of metalloproteinases) variants that are selective metalloproteinase inhibitors. *Biochemical Society Symposium*.

[5] Koskivirta I, Rahkonen O, Mäyränpää M (2006). Tissue inhibitor of metalloproteinases 4 (TIMP4) is involved in inflammatory processes of human cardiovascular pathology. *Histochemistry and Cell Biology*.

[6] Kuroda K, Shinkai H (1997). Gene expression of types I and III collagen, decorin, matrix metalloproteinases and tissue inhibitors of metalloproteinases in skin fibroblasts from patients with systemic sclerosis. *Archives of Dermatological Research*.

[7] Mattila L, Airola K, Ahonen M (1998). Activation of tissue inhibitor of metalloproteinases-3 (TIMP-3) mRNA expression in scleroderma skin fibroblasts. *Journal of Investigative Dermatology*.

[8] Greene J, Wang M, Liu YE, Raymond LA, Rosen C, Shi YE (1996). Molecular cloning and characterization of human tissue inhibitor of metalloproteinase 4. *The Journal of Biological Chemistry*.

[9] Fielitz J, Leuschner M, Zurbrügg HR (2004). Regulation of matrix metalloproteinases and their inhibitors in the left ventricular myocardium of patients with aortic stenosis. *Journal of Molecular Medicine*.

[10] Felkin LE, Birks EJ, George R (2006). A quantitative gene expression profile of matrix metalloproteinases (MMPS) and their inhibitors (TIMPS) in the myocardium of patients with deteriorating heart failure requiring left ventricular assist device support. *The Journal of Heart and Lung Transplantation*.

[11] Stratmann B, Farr M, Tschesche H (2001). MMP-TIMP interaction depends on residue 2 in TIMP-4. *FEBS Letters*.

[12] Williams MH, Handler CE, Akram R (2006). Role of N-terminal brain natriuretic peptide (N-TproBNP) in scleroderma-associated pulmonary arterial hypertension. *European Heart Journal*.

[13] Sfikakis PP, Kyriakidis M, Vergos C (1990). Diffusing capacity of the lung and nifedipine in systemic sclerosis. *Arthritis & Rheumatism*.

[14] Kostopoulos Ch, Koutsikos J, Toubanakis C (2008). Lung scintigraphy with nonspecific human immunoglobulin G (^99m^TC-HIG) in the evaluation of pulmonary involvement in connective tissue diseases: correlation with pulmonary function tests (PFTs) and high-resolution computed tomography (HRCT). *European Journal of Nuclear Medicine and Molecular Imaging*.

[15] Moyssakis I, Gialafos E, Vassiliou V (2005). Aortic stiffness in systemic sclerosis is increased independently of the extent of skin involvement. *Rheumatology*.

[16] Tei C, Dujardin KS, Hodge DO (1996). Doppler echocardiographic index for assessment of global right ventricular function. *Journal of the American Society of Echocardiography*.

[17] McQuillan BM, Picard MH, Leavitt M, Weyman AE (2001). Clinical correlates and reference intervals for pulmonary artery systolic pressure among echocardiographically normal subjects. *Circulation*.

[18] Mukerjee D, St. George D, Knight C (2004). Echocardiography and pulmonary function as screening tests for pulmonary arterial hypertension in systemic sclerosis. *Rheumatology*.

[19] LeRoy EC, Black C, Fleischmajer R (1988). Scleroderma (systemic sclerosis): classification, subsets and pathogenesis. *Journal of Rheumatology*.

[20] MacGregor AJ, Canavan R, Knight C (2001). Pulmonary hypertension in systemic sclerosis: risk factors for progression and consequences for survival. *Rheumatology*.

[21] Giannelli G, Iannone F, Marinosci F, Lapadula G, Antonaci S (2005). The effect of bosentan on matrix metalloproteinase-9 levels in patients with systemic sclerosis-induced pulmonary hypertension. *Current Medical Research and Opinion*.

[22] Trad S, Amoura Z, Beigelman C (2006). Pulmonary arterial hypertension is a major mortality factor in diffuse systemic sclerosis, independent of interstitial lung disease. *Arthritis & Rheumatism*.

[23] Li H, Simon H, Bocan TMA, Peterson JT (2000). MMP/TIMP expression in spontaneously hypertensive heart failure rats: the effect of ACE- and MMP-inhibition. *Cardiovascular Research*.

[24] Seeland U, Kouchi I, Zolk O, Itter G, Linz W, Böhm M (2002). Effect of ramipril and furosemide treatment on interstitial remodeling in post-infarction heart failure rat hearts. *Journal of Molecular and Cellular Cardiology*.

[25] Bonnema DD, Webb CS, Pennington WR (2007). Effects of age on plasma matrix metalloproteinases (MMPs) and tissue inhibitor of metalloproteinases (TIMPs). *Journal of Cardiac Failure*.

[26] Redfield MM, Rodeheffer RJ, Jacobsen SJ, Mahoney DW, Bailey KR, Burnett JC (2002). Plasma brain natriuretic peptide concentration: impact of age and gender. *Journal of the American College of Cardiology*.

[27] Spencer KT, Kirkpatrick JN, Mor-Avi V, Decara JM, Lang RM (2004). Age dependency of the Tei index of myocardial performance. *Journal of the American Society of Echocardiography*.

[28] Allanore Y, Borderie D, Avouac J (2008). High N-terminal pro-brain natriuretic peptide levels and low diffusing capacity for carbon monoxide as independent predictors of the occurrence of precapillary pulmonary arterial hypertension in patients with systemic sclerosis. *Arthritis & Rheumatism*.

[29] Toubi E, Kessel A, Grushko G, Sabo E, Rozenbaum M, Rosner I (2002). The association of serum matrix metalloproteinases and their tissue inhibitor levels with scleroderma disease severity. *Clinical and Experimental Rheumatology*.

[30] Yazawa N, Kikuchi K, Ihn H (2000). Serum levels of tissue inhibitor of metalloproteinases 2 in patients with systemic sclerosis. *Journal of the American Academy of Dermatology*.

[31] Giannelli G, Iannone F, Marinosci F, Lapadula G, Antonaci S (2006). Clinical outcomes of bosentan in pulmonary arterial hypertension do not correlate with levels of TIMPs. *European Journal of Clinical Investigation*.

[32] Dziankowska-Bartkowiak B, Waszczykowska E, Zalewska A, Sysa-Jędrzejowska A (2005). Correlation of endostatin and tissue inhibitor of metalloproteinases 2 (TIMP2) serum levels with cardiovascular involvement in systemic sclerosis patients. *Mediators of Inflammation*.

[33] Shahin A, Elsawaf A, Ramadan S, Shaker O, Amin M, Taha M (2006). Serum levels of tissue inhibitors of metalloproteinase 2 in patients with systemic sclerosis with duration more than 2 years: correlation with cardiac and pulmonary abnormalities. *Mediators of Inflammation*.

[34] Young-Min SA, Beeton C, Laughton R (2001). Serum TIMP-1, TIMP-2, and MMP-1 in patients with systemic sclerosis, primary Raynaud's phenomenon, and in normal controls. *Annals of the Rheumatic Diseases*.

[35] Kim W-U, Min S-Y, Cho M-L (2005). Elevated matrix metalloproteinase-9 in patients with systemic sclerosis. *Arthritis Research & Therapy*.

[36] Sfikakis PP, McCune BK, Tsokos M, Aroni K, Vayiopoulos G, Tsokos GC (1993). Immunohistological demonstration of transforming growth factor-*β* isoforms in the skin of patients with systemic sclerosis. *Clinical Immunology and Immunopathology*.

[37] Verrecchia F, Laboureau J, Verola O (2007). Skin involvement in scleroderma: where histological and clinical scores meet. *Rheumatology*.

[38] Han Y-P, Tuan T-L, Hughes M, Wu H, Garner WL (2001). Transforming growth factor-*β*- and tumor necrosis factor-*α* -mediated induction and proteolytic activation of MMP-9 in human skin. *The Journal of Biological Chemistry*.

[39] Schermuly RT, Kreisselmeier KP, Ghofrani HA (2004). Antiremodeling effects of iloprost and the dual-selective phosphodiesterase 3/4 inhibitor tolafentrine in chronic experimental pulmonary hypertension. *Circulation Research*.

[40] Bou-Gharios G, Osman J, Black C, Olsen I (1994). Excess matrix accumulation in scleroderma is caused partly by differential regulation of stromelysin and TIMP-1 synthesis. *Clinica Chimica Acta*.

[41] Vieillard-Baron A, Frisdal E, Eddahibi S (2000). Inhibition of matrix metalloproteinases by lung TIMP-1 gene transfer or doxycycline aggravates pulmonary hypertension in rats. *Circulation Research*.

[42] Briest W, Homagk L, Baba HA (2004). Cardiac remodeling in erythropoietin-transgenic mice. *Cellular Physiology and Biochemistry*.

[43] Tummalapalli CM, Heath BJ, Tyagi SC (2001). Tissue inhibitor of metalloproteinase-4 instigates apoptosis in transformed cardiac fibroblasts. *Journal of Cellular Biochemistry*.

[44] Fernández CA, Moses MA (2006). Modulation of angiogenesis by tissue inhibitor of metalloproteinase-4. *Biochemical and Biophysical Research Communications*.

[45] Seeland U, Haeuseler C, Hinrichs R (2002). Myocardial fibrosis in transforming growth factor-*β*1 (TGF-*β*1) transgenic mice is associated with inhibition of interstitial collagenase. *European Journal of Clinical Investigation*.

[46] Harley ITW, Kaufman KM, Guthridge JM (2007). Whole genome association by DNA pooling leads to interesting candidate genes for systemic sclerosis. *Arthritis & Rheumatism*.

[47] Sfikakis PP, Tsokos GC (1995). Lymphocyte adhesion molecules in autoimmune rheumatic diseases: basic issues and clinical expectations. *Clinical and Experimental Rheumatology*.

